# Association Between Sarcopenia and the Long‐Term Risk of Intervertebral Disc Degeneration

**DOI:** 10.1002/jcsm.70086

**Published:** 2025-10-14

**Authors:** Jianan Chen, Tongzhou Liang, Wenjun Hu, Nianchun Liao, Zaoqiang Zhang, Huihong Shi, Song Liu, Junquan Liang, Yanbo Chen, Youxi Lin, Xianjian Qiu, Dongsheng Huang, Anjing Liang, Wenjie Gao

**Affiliations:** ^1^ Department of Orthopedics Sun Yat‐Sen Memorial Hospital Guangzhou China; ^2^ The Brain Cognition and Brain Disease Institute, Shenzhen Institute of Advanced Technology Chinese Academy of Sciences Shenzhen China; ^3^ Department of Sports Medicine Peking University Shenzhen Hospital Shenzhen China; ^4^ Shenzhen Bao'an Chinese Medicine Hospital, The Seventh Clinical Medical School of Guangzhou University of Chinese Medicine Shenzhen Guangdong China

**Keywords:** grip strength, intervertebral disc degeneration, sarcopenia, UK Biobank

## Abstract

**Background:**

Sarcopenia and intervertebral disc degeneration (IDD) are both highly prevalent among the elderly and have a substantial impact on their quality of life. However, the association between sarcopenia and IDD remains unclear. This study aimed to investigate whether sarcopenia is independently associated with an increased risk of IDD in middle‐aged and older adults, using prospective data from the UK Biobank.

**Methods:**

A total of 378 773 participants from the UK Biobank were included and categorized into three groups based on the European Working Group on Sarcopenia in Older People 2 (EWGSOP2) criteria: normal, probable sarcopenia and confirmed sarcopenia. The association between sarcopenia and IDD was examined using Kaplan–Meier survival analysis and Cox proportional hazards models. Sensitivity analyses included subgroup analyses to assess the robustness of findings and interaction tests to explore potential effect modifiers.

**Results:**

The median age of participants was 59 years, with females accounting for 54.8% of the cohort. Over a median follow‐up duration of 171 months, 10 585 participants developed IDD. In unadjusted Cox regression analyses, compared to the normal group, the hazard ratios (HRs) for IDD were 1.51 (95% CI: 1.41–1.61) in the probable sarcopenia group and 1.47 (95% CI: 1.14–1.90) in the confirmed sarcopenia group. After adjusting for multiple covariates, the corresponding HRs were 1.35 (95% CI: 1.26–1.44) and 1.41 (95% CI: 1.10–1.80), respectively. These associations remained consistent across subgroup analyses. Notably, in BMI‐stratified analyses, individuals with sarcopenia and a BMI > 25 had a higher risk of IDD (HR: 1.88; 95% CI: 1.31–2.71) compared to those with BMI ≤ 25 (HR: 1.51; 95% CI: 1.06–2.16), with a significant interaction (*p* < 0.001).

**Conclusions:**

Sarcopenia is associated with an increased risk of IDD, particularly in overweight or obese individuals. Regular assessment of muscle strength and mass, along with promoting physical activity and adequate nutritional interventions in ageing populations, may help prevent sarcopenia and delay the onset of IDD.

## 1 Introduction

Intervertebral disc degeneration (IDD) is a common musculoskeletal degenerative disease, considered as a major contributor to low‐back pain (LBP) [1]. Discogenic LBP is the most common type of chronic LBP, accounting for 39% of chronic LBP cases [2]. The pathogenesis of IDD involves the gradual loss of proteoglycans and hydration caused by a long‐term imbalance between the anabolic and catabolic processes of the disc, leading to changes in its composition [3]. Eventually, this process leads to osteophyte formation, endplate sclerosis and reduced disc height. Sarcopenia refers to the age‐related decline in skeletal muscle mass (SMM), muscle strength or physical function [4]. Its prevalence increases with age and has been shown to be associated with falls, fractures, physical disability and mortality [5]. With the worsening of population ageing, sarcopenia is becoming a growing social and healthcare burden (Data S1: [S1]). The incidence of sarcopenia in the elderly population in Europe is between 4.6% and 7.9% [6]. Furthermore, sarcopenia is also associated with an increased mortality rate [5]. A previous meta‐analysis study investigated the relationship between sarcopenia and the risk of mortality in adults; the result suggested that the risk of death in the sarcopenia group was twice that of the normal group [7]. Emerging evidence suggests that sarcopenia is associated with degenerative spinal diseases, such as degenerative scoliosis and lumbar spinal stenosis [8, 9]. Yan et al. compared the muscle conditions of 87 middle‐aged and elderly women over the age of 40 with degenerative lumbar scoliosis and 884 healthy subjects [10]. They found that the prevalence of sarcopenia was significantly higher in women with degenerative scoliosis than in the control group [9, 10]. The prevalence of sarcopenia is also higher in patients with lumbar spinal stenosis [8].

IDD and sarcopenia are both common diseases in the elderly, but the relationship between the two disorders remains unclear. A Japanese cohort demonstrated the potential association between musculoskeletal diseases and lumbar disc degeneration using health examination data from 276 participants, finding no significant association between sarcopenia and disc degeneration at any level [11]. Qi et al. conducted a two‐sample Mendelian randomization analysis and found that appendicular lean mass was positively correlated with other intervertebral disc disorders and disc herniation or disc protrusion (POSD), while grip strength was positively correlated with POSD [6]. However, to date, published studies on the relationship between sarcopenia and IDD have mainly relied on cross‐sectional studies, were limited to Asian populations, and had relatively small sample sizes. A large‐scale population study is needed to determine the relationship between IDD and sarcopenia.

The UK Biobank is a prospective cohort study that collected genetic information and blood samples, as well as lifestyle and environmental exposure data from 500 000 volunteers aged 40–69, and followed their health records over the subsequent decades to explore relationships between specific genes, lifestyle factors and health conditions. Therefore, this study aims to use UK Biobank data to determine whether sarcopenia is independently associated with an increased risk of disc degeneration in middle‐aged and older adults, in order to find more measures to prevent IDD.

## 2 Method

### 2.1 Study Participants and Data Source

The data used in this prospective cohort study came from the UK Biobank, which includes over 500 000 UK participants aged between 40 and 69 at the time of their initial recruitment between 2006 and 2010. Self‐reported health behaviours, physical examinations and biological sample analysis data were collected, and participants were followed up over the long term. Ethical approval was obtained from the Northwest Multi‐Centre Research Ethics Committee and written informed consent was obtained from all participants. This study adheres to the Strengthening the Reporting of Observational Studies in Epidemiology (STROBE) guidelines. Data for this work was obtained from the UK Biobank under the approved application No. 117320. The ethical approval for UK Biobank studies from the NHS National Research Ethics Service on June 17, 2011 (Ref 11/NW/0382) and May 10, 2016 (Ref 16/NW/0274) is applicable for this study.

The inclusion criteria for this study were as follows: (1) Only White individuals were included, as the SMM calculation formula is applicable exclusively to White individuals. (2) Patients who completed the full follow‐up process. (3) Patients who underwent handgrip strength (HGS) measurement, bioelectrical impedance analysis (BIA) and height measurement. The following were excluded: (1) no follow time or no ICD‐10 diagnosis, and participants diagnosed with IDD at baseline; (2) individuals with a history of paralysis, myasthenia gravis or muscular dystrophy; and (3) HGS, BIA and height values were null or unavailable.

### 2.2 Main Exposure Variable and Covariates

Sarcopenia was assessed according to the European Working Group on Sarcopenia in Older People 2 (EWGSOP2) criteria, using HGS and skeletal muscle index (SMI). The HGS test was performed using a Jamar J00105 hydraulic hand dynamometer. Participants were seated with the arm holding the dynamometer positioned at their side, bent at a 90° angle and the forearm resting on the armrest. They were instructed to squeeze the handle of the dynamometer and exert maximum force within 3 s (Data S1: [S2]). The higher value from both hands was selected and expressed in absolute units (kg). If the HGS value is less than 16 kg in women and less than 27 kg in men, it is considered low grip strength. SMI was calculated by dividing SMM (kg) by the square of height (m) [12]. Participants removed their shoes and heavy clothing before testing BIA, which was performed using the Tanita BC418MA (Tanita Europe, Amsterdam, The Netherlands) body composition analyser ([Data S1: S3]). SMM was calculated using the Janssen equation, as follows [13]. The validity of BIA in measuring muscle mass has been previously confirmed and reported.

$$\begin{array}{c} \mathrm{SMM}=\left[\left(\frac{{\text{height}}^{2}}{\mathrm{BIA}}\times 0.401\right)+\left(\text{gender}\times 3.825\right)+\left(\mathrm{age}\times -0.071\right)\right]+5.102 \end{array}$$

Low SMI is defined as being less than 5.5 kg/m^2^ in women and less than 7.0 kg/m^2^ in men. According to the EWGSOP2 criteria, the population is divided into three subgroups: the normal group (normal HGS and normal SMI); the probable sarcopenia group (low HGS and normal SMI; normal HGS and low SMI); and the confirmed sarcopenia group (low HGS and low SMI).

Other covariates included age, gender, body mass index (BMI), heel bone mineral density (BMD) T‐score, Townsend deprivation index, education, smoking status, alcohol status, physical activity, sedentary time and medication history (corticosteroids, statins and calcium supplements). Included comorbidities were heart failure, diabetes, hyperlipidaemia, hypertension and liver disease. BMI was calculated by dividing weight by the square of height, both measured at baseline. Heel bone density was measured at baseline using an ultrasonic bone densitometer. The Townsend deprivation index, education, smoking, alcohol consumption, physical activity and sedentary time were self‐reported by participants. The Townsend deprivation index was an indicator of socioeconomic status, with higher values indicating lower socioeconomic status. Education level was categorized into higher (college/university degree or other professional qualifications) and lower levels. Smoking status was classified as non‐smoker and smoker. Alcohol intake frequency was categorized as infrequent intake (< 3 times/week), moderate intake (3–4 times/week) and frequent intake (> 4 times/week). Physical activity was assessed using weekly Metabolic Equivalent Task (MET) minutes, including walking, moderate and vigorous activities, and low physical activity was defined as less than 500 MET minutes per week (Data S1: [S4]). Sedentary behaviour was defined as the total time spent watching TV, using a computer and driving, with more than 6 h per day classified as high sedentary behaviour (Data S1: [S5]). The medication history was determined based on the questionnaires of the participants at baseline, and the previous medication situation of each participant was recorded in detail. Comorbidities were identified using diagnoses recorded with International Classification of Diseases (ICD) codes, which can be directly retrieved from the UK Biobank database. More detailed information about these measurements can be found on the UK Biobank website (http://www.ukbiobank). Missing data for covariates were imputed using multiple imputation by chained equations (Data S1: [S6]).

### 2.3 Follow Time and Outcome

The follow‐up period started from the day participants completed the baseline survey and continued until the occurrence of IDD, death, loss to follow‐up or the end of the follow‐up period. The last follow‐up date was July 1, 2023, when data collection for this study was completed. The diagnosis of IDD was determined through hospital diagnoses (ICD‐10 codes of G55.1; M51.1; M51.16; M51.19; M51.2; M51.3; M51.37; M51.8; M51.9) [14].

### 2.4 Statistical Analysis

A normality test was performed for each continuous variable, and since they did not follow a normal distribution, continuous variables were described using the median (interquartile range). The Kruskal–Wallis *H* test was used for comparisons. Categorical variables were presented as case numbers (percentages), and group comparisons were performed using the Chi‐square test. The Kaplan–Meier method was used to analyse the effect of sarcopenia on the cumulative incidence of IDD, and the log‐rank test was used to compare differences between groups. A univariate Cox proportional hazards model was used to calculate the relationship between sarcopenia and the incidence of disc degeneration, with results expressed as hazard ratios (HRs) and 95% confidence intervals (95% CI). We designed three multivariate Cox proportional hazards models to adjust for covariates: Model 1 adjusted for gender, age and BMI; Model 2 further adjusted for smoking, alcohol consumption, deprivation index, activity time, sedentary time and education level; Model 3, in addition to adjusting for the above risk factors, further adjusted for comorbidities such as heart failure, diabetes, hyperlipidemia, hypertension and liver disease. To test the robustness of the results, multiple sensitivity analyses were conducted. Subgroup analyses were performed according to age (< 65 years or ≥ 65 years), gender (male or female), education level (higher or lower), Townsend Deprivation Index (Low: < mean or High: > mean), BMI (< 25 or ≥ 25), smoking status (never, former or current), alcohol consumption (infrequent, moderate, frequent), physical inactivity (yes or no), sedentary behaviour (yes or no), various medication histories (yes or no) and various comorbidities (yes or no). Interaction tests were conducted to determine whether there was an interaction between sarcopenia and each covariate. If P for interaction < 0.05, this indicates that there is a difference in HR values between different subgroups.

All statistical analyses were performed in R (version 4.2.2), using the grid, cmprsk, tidyverse, forestplot, dplyr, ggplot2, jstable, mice and survival packages. A two‐tailed *p*‐value < 0.05 was considered statistically significant.

## 3 Results

### 3.1 Baseline Characteristics of Participants

**FIGURE 1:**
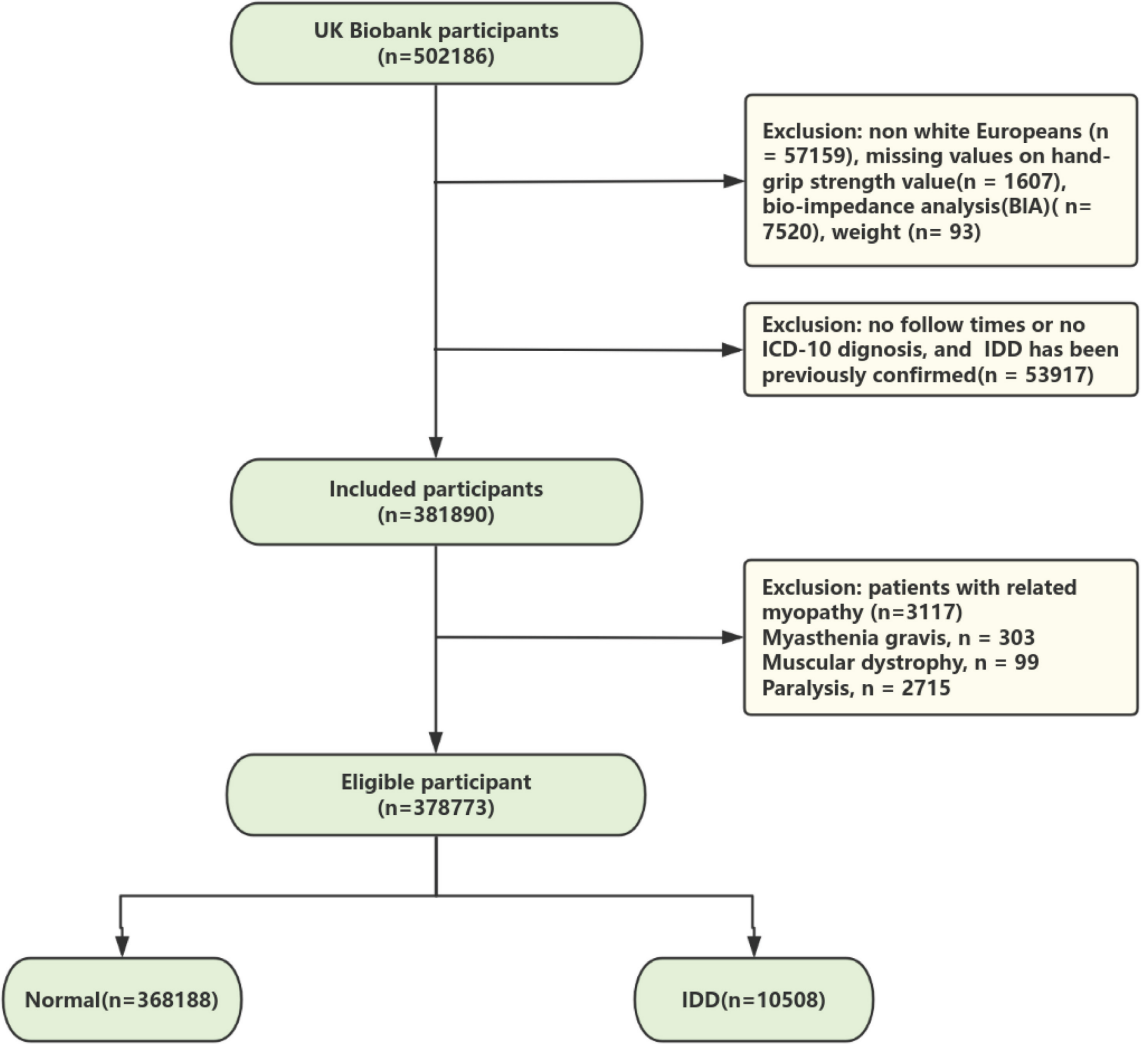
Flow chart of participant selection in this study.

The final analysis included 378 773 participants (median age 59 years, 54.8% female), with a median follow‐up time of 171 months (Figure 1). A total of 10 585 patients developed IDD (Table 1). Compared to participants without IDD, those with IDD were older, had a higher BMI and a higher Townsend deprivation score. They had a lower education level, engaged in less personal activity, exhibited more sedentary behaviour, smoked more and consumed less alcohol. Patients with IDD were more likely to have a history of corticosteroids, statins and calcium supplements. Patients with IDD had more comorbidities, with higher incidence rates of heart failure, diabetes, hyperlipidaemia, hypertension and liver disease compared to the normal group.

**TABLE 1:** Baseline characteristics of participants categorized by non‐IDD and IDD.

Variable	Group	Overall	Non‐IDD	IDD	*p*‐value
N		378 773	368 188	10 585	< 0.001
Age (years)		59.00 [51.00, 64.00]	59.00 [51.00, 64.00]	60.00 [53.00, 65.00]	< 0.001
Sex	Female	207 559 (54.8)	201 422 (54.7)	6137 (58.0)	
	Man	171 214 (45.2)	166 766 (45.3)	4448 (42.0)	
BMI (kg/m^2^)		26.81 [24.20, 29.98]	26.78 [24.18, 29.94]	27.93 [25.07, 31.39]	< 0.001
Sarcopenia	Normal	350 746 (92.6)	341 255 (92.7)	9491 (89.7)	< 0.001
	Probable	26 441 (7.0)	25 407 (6.9)	1034 (9.8)	
	Sarcopenia	1586 (0.4)	1526 (0.4)	60 (0.6)	
Follow up time (months)		171.00 [161.00, 180.00]	171.00 [162.00, 180.00]	95.00 [54.00, 130.00]	< 0.001
Education	Higher	264 719 (69.9)	256 491 (69.7)	8228 (77.7)	< 0.001
	Lower	114 054 (30.1)	111 697 (30.3)	2357 (22.3)	
Heel BMD T score		−0.46 [−1.15, 0.31]	−0.46 [−1.15, 0.31]	−0.44 [−1.13, 0.34]	0.026
Townsend deprivation index		−2.28 [−3.70, 0.21]	−2.29 [−3.71, 0.20]	−2.05 [−3.55, 0.74]	< 0.001
Personal activity	Low	57 388 (15.2)	55 652 (15.1)	1736 (16.4)	< 0.001
	High	321 385 (84.8)	312 536 (84.9)	8849 (83.6)	
Sedentary behaviour	Low	300 919 (79.4)	292 912 (79.6)	8007 (75.6)	< 0.001
	High	77 854 (20.6)	75 276 (20.4)	2578 (24.4)	
Smoking status	Never	202 513 (53.5)	197 553 (53.7)	4960 (46.9)	< 0.001
	Previous	136 564 (36.1)	132 361 (35.9)	4203 (39.7)	
	Current	39 696 (10.5)	38 274 (10.4)	1422 (13.4)	
Alcohol intake frequency	Infrequent	209 596 (55.3)	203 250 (55.2)	6346 (60.0)	< 0.001
	Moderate	89 790 (23.7)	87 610 (23.8)	2180 (20.6)	
	Frequent	79 387 (21.0)	77 328 (21.0)	2059 (19.5)	
Diabetes	No	358 930 (94.8)	349 185 (94.8)	9745 (92.1)	< 0.001
	Yes	19 843 (5.2)	19 003 (5.2)	840 (7.9)	
Hypertension	No	242 448 (64.0)	237 709 (64.6)	4739 (44.8)	< 0.001
	Yes	136 325 (36.0)	130 479 (35.4)	5846 (55.2)	
Hyperlipidemia	No	367 031 (96.9)	357 124 (97.0)	9907 (93.6)	< 0.001
	Yes	11 742 (3.1)	11 064 (3.0)	678 (6.4)	
Heart failure	No	367 612 (97.1)	357 662 (97.1)	9950 (94.0)	< 0.001
	Yes	11 161 (2.9)	10 526 (2.9)	635 (6.0)	
Liver disease	No	375 259 (99.1)	364 854 (99.1)	10 405 (98.3)	< 0.001
	Yes	3514 (0.9)	3334 (0.9)	180 (1.7)	
Corticosteroids	No	374 149 (98.78)	363 789 (98.81)	10 360 (97.87)	< 0.001
	Yes	4624 (1.22)	4399 (1.19)	225 (2.13)	
Statin	No	317 594 (83.85)	309 313 (84.01)	8281 (78.23)	< 0.001
	Yes	61 179 (16.15)	58 875 (15.99)	2304 (21.77)	
Calcium supplements	No	373 618 (98.64)	363 234 (98.65)	10 384 (98.10)	< 0.001
	Yes	5155 (1.36)	4954 (1.35)	201 (1.90)	

*Note:* Variables are presented as median (IQR) or *n* (%).

Abbreviations: BMD = bone mineral density; BMI = body mass index; IDD = intervertebral disc degeneration.

### 3.2 Sarcopenia and Long‐Term Risk of IDD

**FIGURE 2:**
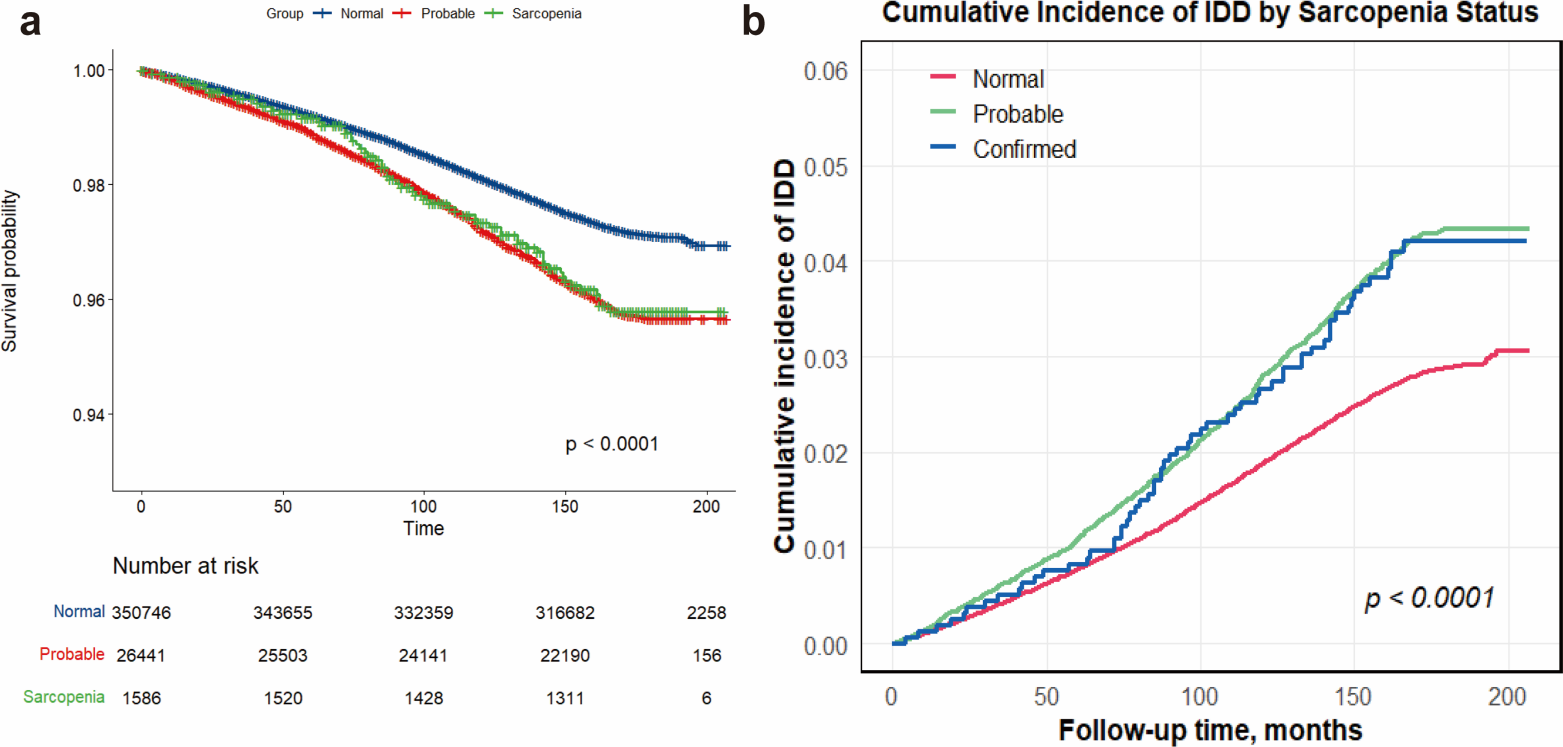
Association between sarcopenia status and the risk of IDD. (a) Kaplan–Meier survival curves for IDD survival stratified by sarcopenia status. The blue line represents the Normal group, the red line represents the probable sarcopenia group and the green line represents the confirmed sarcopenia group. (b) Cumulative incidence curves for IDD by sarcopenia status. The red line represents the Normal group, the green line represents the probable sarcopenia group and the blue line represents the confirmed sarcopenia group. IDD: Intervertebral disc degeneration.

Participants who developed IDD had a higher incidence of sarcopenia compared to the normal group. Among IDD patients, there were 1034 (9.8%) in the probable sarcopenia group and 60 (0.6%) in the confirmed sarcopenia group, while among participants without IDD, there were 25 407 (6.9%) in the probable sarcopenia group and 1526 (0.4%) in the confirmed sarcopenia group. Similarly, survival analysis showed that the cumulative incidence of IDD in the probable sarcopenia group and confirmed sarcopenia groups was higher compared to the non‐sarcopenia group (Figure 2; log‐rank test result *p* < 0.001). Compared to participants with normal GS, those with low GS demonstrated a significantly higher risk of developing IDD (Figure S1). Compared with participants with normal SMM, low SMM did not significantly increase the risk of IDD (Figure S2).

**FIGURE 3:**
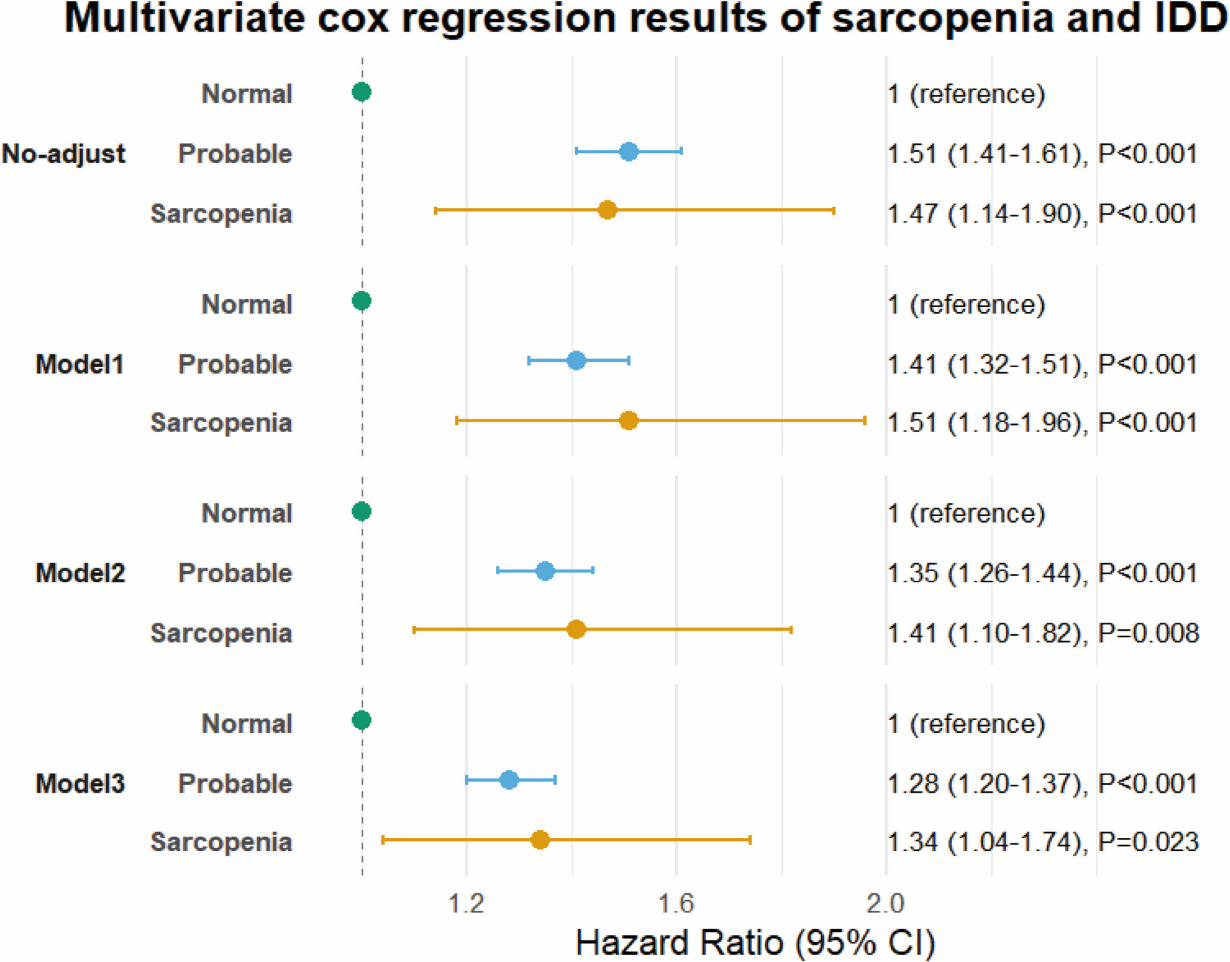
Results of the association analysis between sarcopenia and the risk of IDD occurrence. Model 1 adjusted for gender, age and BMI. Model 2 adjusted for gender, age, BMI, Townsend deprivation index, education level, smoking, alcohol consumption, physical activity time, sedentary time, history of using corticosteroids, statins and calcium supplements. Model 3, in addition to adjusting for the above variables, also adjusted for heart failure, diabetes, hyperlipidemia, hypertension and liver disease. IDD: Intervertebral disc degeneration.

With the non‐sarcopenia group as the reference, the univariate Cox regression results indicated that the HRs for the incidence of IDD in the probable sarcopenia group and the confirmed sarcopenia group were 1.51 (1.41–1.61) and 1.47 (1.14–1.90), respectively (Figure 3). We further adjusted for covariates; in Cox proportional hazards Model 1, after adjusting for gender, age and BMI, the HRs for the two groups were 1.41 (1.32–1.51) and 1.51 (1.18–1.96), respectively. In Model 2, after further adjusting for deprivation index, education level, smoking, alcohol consumption, physical activity, sedentary time and medication history, the HRs for the two groups remained 1.35 (1.26–1.44) and 1.41 (1.10–1.82). In addition to the above variables, Model 3 continued to adjust for comorbidities, with HRs for the two groups being 1.28 (95% CI, 1.20–1.37) and 1.34 (95% CI, 1.04–1.74), respectively. After adjusting for all covariates, low grip strength is associated with an increased risk of IDD (HR = 1.44, 95% CI: 1.34–1.55, *p* < 0.001) (Figure S3). There was no statistically significant risk associated with low SMM and the occurrence of IDD (HR = 0.93, 95% CI: 0.86–1.00, *p* = 0.06) after adjusting for all covariates (Figure S4).

### 3.3 Subgroup Analysis and Interaction Tests

In the subgroup analysis, due to the small number of IDD patients with liver disease, the HR for the confirmed sarcopenia group in this subgroup could not be estimated. The remaining results showed that in most subgroups, the probable sarcopenia group and the confirmed sarcopenia group were associated with the occurrence of IDD (Table 2). Interaction tests indicated that participants with a BMI > 25 had a significantly higher risk of developing IDD compared to those with a BMI ≤ 25 (P for interaction < 0.001). In the confirmed sarcopenia group, the HR for IDD occurrence in patients with a BMI > 25 was 1.88 (1.31–2.71), while the HR for IDD occurrence in patients with a BMI ≤ 25 was 1.51 (1.06–2.16). No interactions were found in other subgroups during the interaction tests.

**TABLE 2:** Subgroup analysis and interaction test results for sarcopenia and IDD Incidence.

		Sarcopenia			
Subgroup	Count	Normal	Probable	Confirmed	P for interaction
Sex					0.471
Female	207 559	1 (reference)	1.46 (1.35 to 1.57)	1.39 (1.07 to 1.81)	
Male	171 214	1 (reference)	1.56 (1.38 to 1.76)	2.37 (0.76 to 7.34)	
Age (years)					0.402
> 65	60 922	1 (reference)	1.36 (1.21 to 1.53)	1.29 (0.86 to 1.95)	
< = 65	317 851	1 (reference)	1.48 (1.37 to 1.60)	1.44 (1.04 to 1.99)	
BMI (kg/m^2^)					**<0.001**
> 25	255 801	1 (reference)	1.80 (1.67 to 1.94)	1.88 (1.31 to 2.71)	
< = 25	122 972	1 (reference)	1.14 (1.00 to 1.29)	1.51 (1.06 to 2.16)	
Education					0.596
Lower	264 719	1 (reference)	1.47 (1.37 to 1.58)	1.36 (1.03 to 1.79)	
Higher	114 054	1 (reference)	1.38 (1.17 to 1.62)	1.74 (0.93 to 3.23)	
Smoking status					0.825
Non‐smoker	202 513	1 (reference)	1.49 (1.36 to 1.63)	1.41 (0.98 to 2.03)	
Smoker	176 260	1 (reference)	1.53 (1.40 to 1.67)	1.58 (1.11 to 2.25)	
Alcohol intake frequency					0.336
Infrequent	209 596	1 (reference)	1.52 (1.41 to 1.65)	1.24 (0.90 to 1.71)	
Moderate	89 790	1 (reference)	1.36 (1.16 to 1.60)	2.07 (1.17 to 3.65)	
Frequent	79 387	1 (reference)	1.45 (1.24 to 1.70)	1.84 (1.01 to 3.32)	
Personal activity					0.582
Low	57 388	1 (reference)	1.59 (1.38 to 1.84)	1.30 (0.72 to 2.36)	
High	321 385	1 (reference)	1.48 (1.38 to 1.59)	1.51 (1.14 to 2.00)	
Sedentary time					0.466
Low	300 919	1 (reference)	1.48 (1.37 to 1.59)	1.54 (1.17 to 2.04)	
High	77 854	1 (reference)	1.60 (1.41 to 1.82)	1.26 (0.70 to 2.29)	
Townsend deprivation index					0.209
Low	278 409	1 (reference)	1.43 (1.32 to 1.55)	1.43 (1.05 to 1.95)	
High	100 364	1 (reference)	1.61 (1.45 to 1.79)	1.56 (1.00 to 2.42)	
Hypertension					0.868
No	242 448	1 (reference)	1.37 (1.23 to 1.52)	1.36 (0.91 to 2.04)	
Yes	136 325	1 (reference)	1.41 (1.31 to 1.53)	1.47 (1.06 to 2.04)	
Diabetes					0.761
No	358 930	1 (reference)	1.48 (1.39 to 1.59)	1.46 (1.12 to 1.90)	
Yes	19 843	1 (reference)	1.51 (1.24 to 1.84)	2.18 (0.82 to 5.83)	
Heart failure					0.113
No	367 612	1 (reference)	1.50 (1.40 to 1.60)	1.42 (1.08 to 1.85)	
Yes	11 161	1 (reference)	1.21 (0.97 to 1.53)	2.11 (0.94 to 4.72)	
Liver disease					0.286
No	375 259	1 (reference)	1.50 (1.41 to 1.60)	1.50 (1.16 to 1.94)	
Yes	3514	1 (reference)	1.43 (0.94 to 2.19)	—	
Hyperlipidemia					0.505
No	367 031	1 (reference)	1.48 (1.39 to 1.59)	1.52 (1.18 to 1.97)	
Yes	11 742	1 (reference)	1.53 (1.22 to 1.91)	0.74 (0.18 to 2.95)	
Corticosteroids					0.659
No	374 149	1 (reference)	1.50 (1.40 to 1.60)	1.42 (1.09 to 1.85)	
Yes	4624	1 (reference)	1.30 (0.93 to 1.82)	1.73 (0.71 to 4.20)	
Statin					0.478
No	317 594	1 (reference)	1.47 (1.36 to 1.58)	1.35 (1.00 to 1.82)	
Yes	61 179	1 (reference)	1.48 (1.31 to 1.68)	1.93 (1.20 to 3.11)	
Calcium supplements					0.532
No	373 618	1 (reference)	1.49 (1.40 to 1.60)	1.51 (1.16 to 1.95)	
Yes	7777	1 (reference)	1.48 (1.07 to 2.05)	0.81 (0.26 to 2.54)	

Abbreviations: BMI = body mass index.

## 4 Discussion

In this study, we conducted a prospective cohort study using the UK Biobank database to explore the relationship between sarcopenia and IDD. We found that, after adjusting for multiple factors, participants who were probable sarcopenia or were confirmed sarcopenia were associated with a long‐term high risk of developing IDD. This association remained evident in multiple sensitivity analyses. Additionally, we observed that sarcopenic participants who were obese were more likely to develop IDD than those who were not obese.

Our study findings are consistent with a previous Mendelian randomization study, which revealed a positive correlation between decreased limb muscle mass or grip strength and the development of IDD [6]. Intervertebral discs play a crucial role in intervertebral connectivity and spinal motion. In addition to causing low back pain, IDD can lead to secondary conditions, including spinal stenosis, segmental instability of the spine, disc herniation and compression of the spinal cord and nerve roots. Sarcopenia has been shown to be associated with various degenerative spinal diseases, such as lumbar spinal stenosis, spondylolisthesis, spine fractures and degenerative spinal deformities [8, 15, 16]. The back muscles support the spine to maintain optimal posture and enable mobility, exerting load on the vertebrae [17]. When the strength of the major lumbar muscles (the erector spinae and multifidus) is severely reduced, nearly all other muscles must increase their activity to achieve muscular co‐contraction [18, 19]. This impairs spinal balance and increases the load on the spine [18]. Therefore, functional loss caused by trunk muscle degeneration may increase the risk of vertebral fractures either directly through segmental overload or indirectly by affecting balance [17]. The activity of kyphoscoliosis (Ky) protein is essential for normal muscle growth and function, as well as for the maturation and stabilization of neuromuscular junctions. Mutations in the Ky gene can lead to muscle dysfunction, which may subsequently result in scoliosis. Sarcopenia leads to reduced strength of the paraspinal muscles, which may result in a redistribution of biomechanical loads onto the spine, thereby increasing the risk of degenerative changes (Data S1: [S7]). Additionally, sarcopenia affects the outcomes of degenerative lumbar surgery. Compared to patients without sarcopenia, those with sarcopenia undergoing surgical treatment for degenerative lumbar disease show less improvement in quality of life, functional capacity, pain relief and experience longer hospital stays (Data S1: [S8]). Wu et al. conducted a meta‐analysis exploring the prevalence of sarcopenia and its impact on clinical outcomes of lumbar degenerative spine diseases; however, this study did not confirm that the occurrence of sarcopenia increased the risk of lumbar degenerative spine diseases [20]. The diagnostic criteria for sarcopenia in the studies they included were different, and not all studies assessed both muscle mass and grip strength and therefore may interfere with the conclusion. Furthermore, only two studies included subjects without lumbar lesions as a control group, which may have led to biased results [20]. Compared to previous studies, we included a larger sample size and employed a prospective design. After adjusting for multiple covariates and other comorbidities, we found that the HRs for developing IDD in the probable sarcopenia group and the confirmed sarcopenia group compared to the normal group were 1.28 (95% CI, 1.20–1.37) and 1.34 (95% CI, 1.04–1.74), respectively.

The trunk and paravertebral muscles are composed of skeletal muscle fibres, which play a crucial role in providing stability and support to the spine [16]. Skeletal muscle undergoes atrophy with ageing, with a loss of muscle fibre volume reaching up to 40% from ages 25 to 80 (Data S1: [S9]). Hormones essential for maintaining skeletal muscle mass, such as growth hormone, insulin‐like growth factor and androgens, also decrease in older age (Data S1: [S10]). Additionally, as people age, there is an increase in fat cells and fat deposits within skeletal muscle, processes that ultimately lead to a decline in muscle strength and function. This decline can result in structural instability, potentially contributing to IDD [15, 16]. A previous biomechanical study indicated that as the volume of paravertebral muscles increases, the load on the spine and intervertebral discs decreases. Increasing the volume of paravertebral muscles through exercise can aid in the management of spinal pain and the prevention of IDD [21]. There are existing studies on the mechanisms underlying the relationship between sarcopenia and IDD. Huang et al. found that the occurrence of IDD is directly associated with paraspinal muscle fat infiltration in rats, which may be related to inflammation [2]. They detected the expression of tumour necrosis factor (TNF)‐α in both the intervertebral discs and paraspinal muscles of rats with discogenic LBP [2]. TNF is known for its strong pro‐inflammatory activity and is closely associated with the development of IDD [22]. James et al. collected muscle and fat tissue samples from patients with disc herniation and performed quantitative polymerase chain reaction (qPCR) to analyse gene expression [23]. They found that individuals with high fat infiltration exhibited elevated TNF expression in the multifidus muscle [23]. Shi et al. suggested that the expression of TNF is significantly higher in severely degenerated intervertebral discs compared to mildly degenerated discs [24]. This study found that low HGS was significantly associated with an increased risk of IDD, whereas low SMM did not significantly increase the risk of IDD. This suggests that during the progression of sarcopenia, the onset of reduced grip strength should serve as a warning sign for the potential development of IDD. Higher muscle strength helps maintain spinal stability and reduces spinal degeneration, while the loss of paraspinal muscle strength may increase the load on the lower lumbar intervertebral discs, posterior spinal elements and vertebral bodies, thereby accelerating disc degeneration [21, 25].

In sensitivity analyses, we found that obesity may be a risk factor promoting IDD in sarcopenic patients. Compared to non‐obese patients, obese patients were more likely to experience IDD associated with sarcopenia. Adipose tissue is a highly inflammatory tissue, and the accumulation and polarization of macrophages within it are common features of various health conditions [23, 26]. In obesity and sarcopenia, macrophages in adipose tissue typically shift from the anti‐inflammatory M2 phenotype to the pro‐inflammatory M1 phenotype [26, 27]. James et al. found that in individuals with a high degree of fat infiltration, the expression of Nos2, a marker of M1 macrophages, was elevated in epidural adipose tissue, while the expression of Arg‐1, a marker of M2 macrophages, was reduced in intramuscular and subcutaneous adipose tissue [23]. Furthermore, previous animal studies have shown that in models of IDD, the number of macrophages increases in paraspinal muscles, with a polarization towards the pro‐inflammatory M1 phenotype in both the adipose tissue of these muscles and the nucleus pulposus [28, 29]. Therefore, the imbalance between M1 and M2 macrophages observed in obesity and sarcopenia may play a significant role in the development of IDD. Obesity can increase the load on the vertebrae, leading to more severe degeneration of the lumbar intervertebral discs [30]. Previous studies have shown that patients with sarcopenic obesity exhibit more pronounced declines in walking ability, quality of life and self‐sufficiency [31, 32]. They tend to have lower physical activity levels and spend more time in sedentary behaviours, which may accelerate IDD (Data S1: [S11]). Additionally, obese individuals often exhibit abnormal blood lipid levels, and hypertriglyceridemia can contribute to the IDD [33]. At the molecular level, obesity is considered a pro‐inflammatory state that promotes the release of cytokines such as TNF‐α and IL‐6, thereby facilitating the development of IDD [22]. Therefore, the findings of this study suggest that for patients with sarcopenia, reducing high‐fat dietary intake and body weight may help prevent IDD to some extent.

Sarcopenia may represent a modifiable risk factor for IDD. The prevention of sarcopenia requires a multifaceted approach, including nutrition, exercise and the use of anabolic agents. The preferred preventive approach is primarily based on exercise and nutritional supplementation. Low‐intensity resistance training can induce increases in muscle strength [34]. Aerobic exercise promotes mitochondrial biogenesis and may enhance muscle hypertrophy and strength [35, 36]. The combination of exercise and proper nutrition can stimulate mitochondrial biogenesis and function, increase the number and function of satellite cells, and suppress inflammatory cytokines, resulting in increased protein synthesis and reduced protein degradation [37, 38]. Vitamin D supplementation alongside resistance training can further improve muscle strength [34]. For sarcopenic patients over 65 years of age, the recommended daily protein intake is 1–1.2 g/kg/d (Data S1: [S12]). Studies have shown that testosterone replacement therapy can be used to treat sarcopenia, as testosterone activates protein synthesis and inhibits the differentiation of preadipocyte progenitor cells (Data S1: [S13]). However, the use of testosterone can have side effects, including gynecomastia, erythrocytosis and sleep apnea, and may increase the risk of prostate cancer [39]. Therefore, a comprehensive assessment of the benefits and risks is necessary.

Our study has several superiorities compared with previous studies. It is a large‐scale prospective cohort study with over 370 000 participants and a sufficiently long follow‐up period, with a median follow‐up time of over 14 years, which is beneficial for observing the outcomes of chronic diseases. The impact of sarcopenia on IDD is still unclear, and this study provides robust evidence for the association between the two, guiding the prevention of disc degeneration. However, there are also certain limitations to our study. First, the number of patients with sarcopenia was relatively small, preventing further analysis of those with severe sarcopenia. Second, the covariates included in this study were obtained from baseline surveys and did not account for changes in covariates during the follow‐up period, which may have influenced the results to some extent. Third, although we adjusted for as many covariates as possible, the observed outcomes may still be affected by other confounding factors. For example, we only included data from White people. Fourth, this is a prospective observational study and cannot establish a causal relationship between sarcopenia and IDD. Further randomized controlled trials (RCTs) are needed to clarify this causal link. Lastly, as thoracolumbar MRI data were not available for all participants in the UK Biobank database, this study followed previous literature and used ICD‐10 diagnostic codes to assess the diagnosis of IDD [14]. This approach may have resulted in some cases of IDD being missed.

## 5 Conclusions

In summary, this prospective study provides new evidence for the longitudinal relationship between sarcopenia and IDD. The study revealed that probable sarcopenia or confirmed sarcopenia was associated with a long‐term high risk of developing IDD. Preventing or delaying sarcopenia may help reduce the incidence of IDD. Regular muscle health and muscle strength assessments should be conducted for the elderly. Encourage the elderly to participate in physical exercise and maintain daily essential nutrition, especially for obese patients. Further research that focuses on the potential mechanism underlying our findings is needed.

## Conflicts of Interest

The authors declare no conflicts of interest.

## Supporting information


**Figure S1:** Association between grip strength and the risk of IDD. (a) Kaplan–Meier survival curves for IDD survival stratified by grip strength. The red line represents the low grip strength group, and the blue line represents the normal group. (b) Cumulative incidence curves for IDD by grip strength. The red line represents the normal group, and the light‐blue line represents the low grip strength group. GS: Grip strength; IDD: Intervertebral disc degeneration.

**Figure S2:** Association between SMM and the risk of IDD. (a) Kaplan–Meier survival curves for IDD survival stratified by SMM. The blue line represents the low SMM group, and the red line represents the normal group. (b) Cumulative incidence curves for IDD by SMM. The red line represents the Normal group, and the light‐blue line represents the low SMM group. SMM: skeletal muscle mass; IDD: Intervertebral disc degeneration.

**Figure S3:** Results of the association analysis between grip strength and the risk of IDD occurrence. Model 1 adjusted for gender, age and BMI. Model 2 adjusted for gender, age, BMI, Townsend deprivation index, education level, smoking, alcohol consumption, physical activity time, sedentary time, history of using corticosteroids, statins and calcium supplements. Model 3: in addition to adjusting for the above variables, also adjusted for heart failure, diabetes, hyperlipidaemia, hypertension and liver disease. GS: Grip strength; IDD: Intervertebral disc degeneration.

**Figure S4:** Results of the association analysis between SMM and the risk of IDD occurrence. Model 1 adjusted for gender, age and BMI. Model 2 adjusted for gender, age, BMI, Townsend deprivation index, education level, smoking, alcohol consumption, physical activity time, sedentary time, history of using corticosteroids, statins and calcium supplements. Model 3: in addition to adjusting for the above variables, also adjusted for heart failure, diabetes, hyperlipidaemia, hypertension and liver disease. SMM: skeletal muscle mass; IDD: Intervertebral disc degeneration.

**Data S1:** Supplementary references.
