# Supplementary figures and images for: Association Between Sarcopenia and the Long‐Term Risk of Intervertebral Disc Degeneration
Source: J Cachexia Sarcopenia Muscle. 2025 Oct 14;16(5):e70086. doi: 10.1002/jcsm.70086 (PMC12519499; doi:10.1002/jcsm.70086)

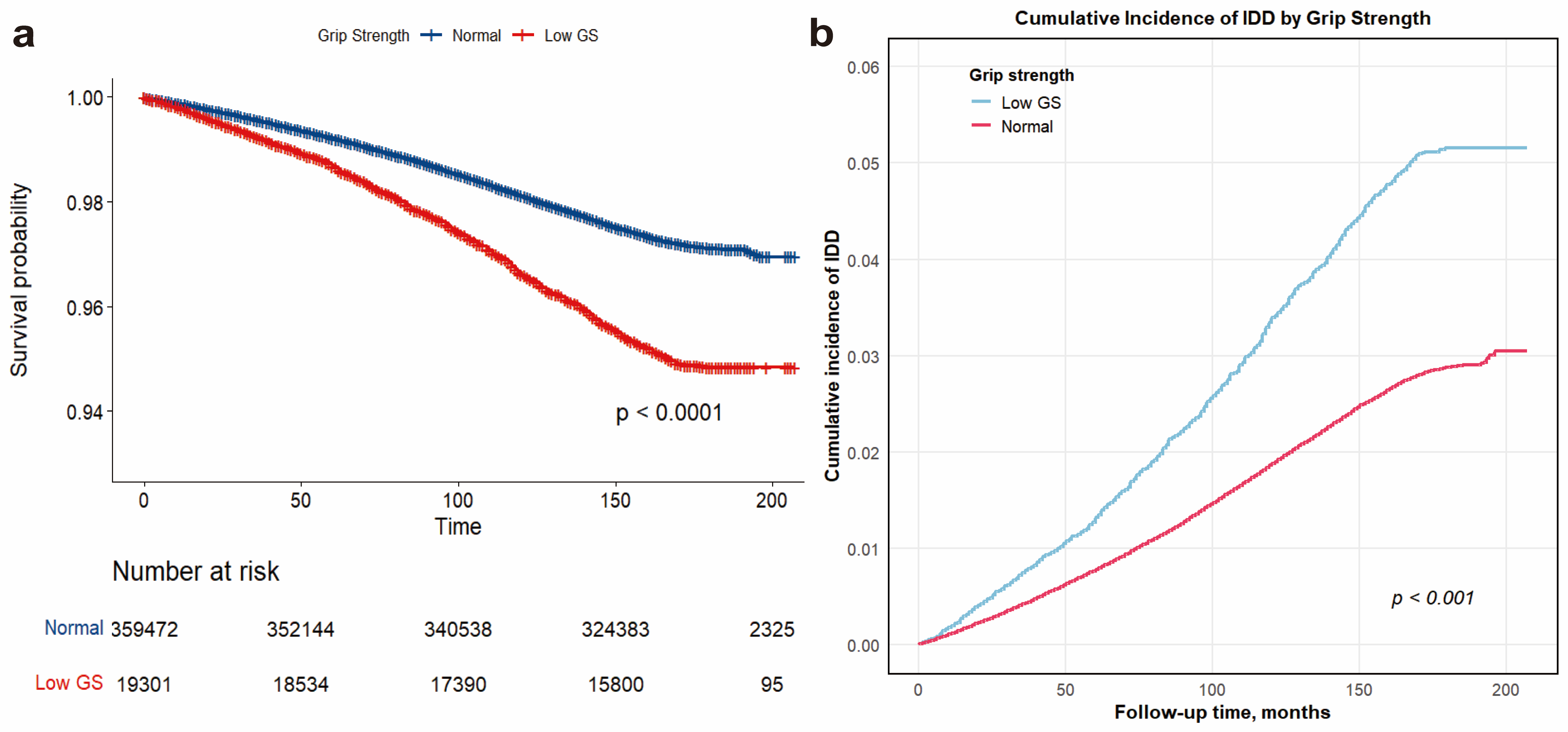

Supplement: Supplementary file 1 — Figure S1: Association between grip strength and the risk of IDD. (a) Kaplan–Meier survival curves for IDD survival stratified by grip strength. The red line represents the low grip strength group, and the blue line represents the normal group. (b) Cumulative incidence curves for IDD by grip strength. The red line represents the normal group, and the light‐blue line represents the low grip strength group. GS: Grip strength; IDD: Intervertebral disc degeneration. [file JCSM-16-e70086-s005.tif]

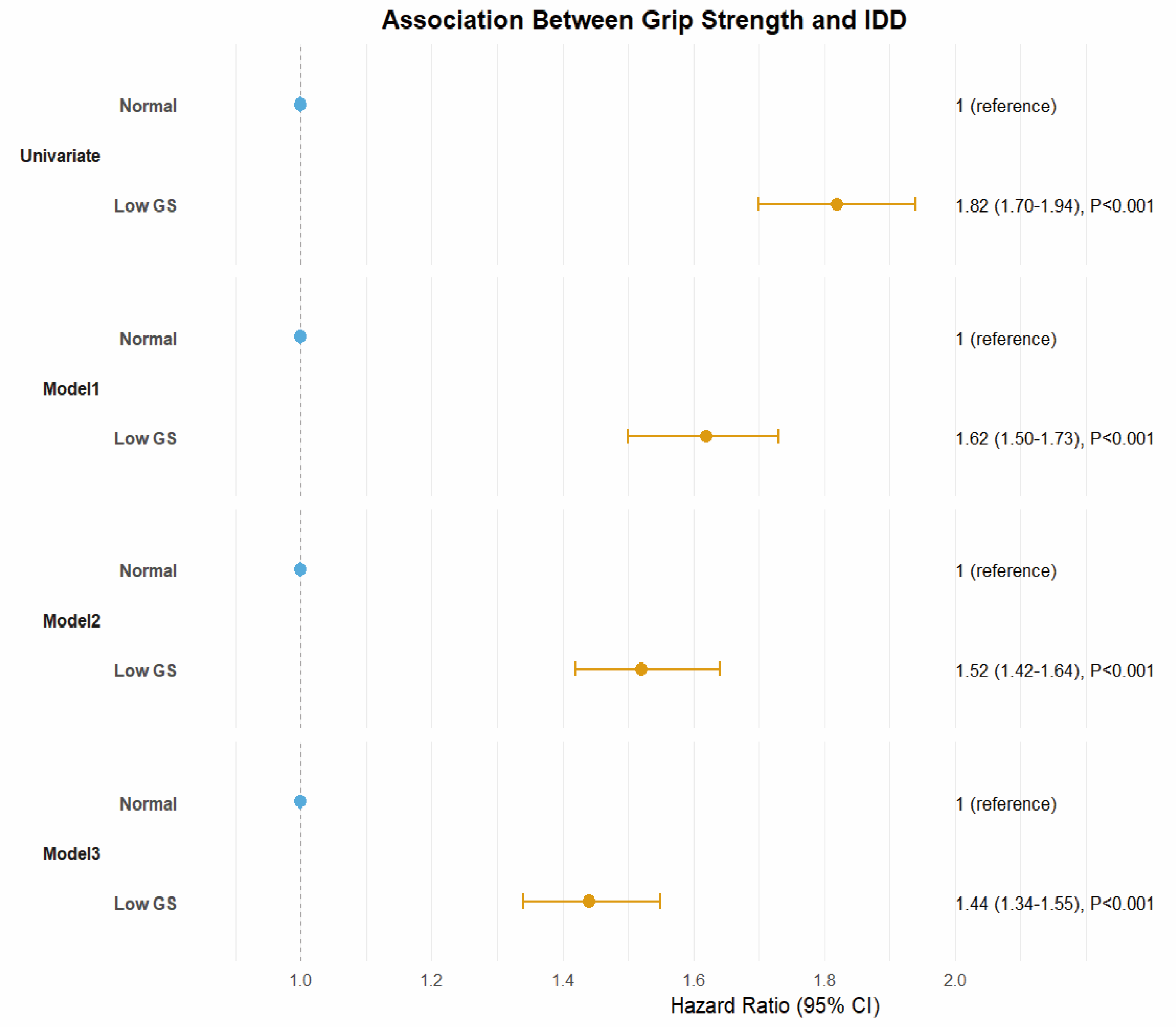

Supplement: Supplementary file 2 — Figure S2: Association between SMM and the risk of IDD. (a) Kaplan–Meier survival curves for IDD survival stratified by SMM. The blue line represents the low SMM group, and the red line represents the normal group. (b) Cumulative incidence curves for IDD by SMM. The red line represents the Normal group, and the light‐blue line represents the low SMM group. SMM: skeletal muscle mass; IDD: Intervertebral disc degeneration. [file JCSM-16-e70086-s004.tif]

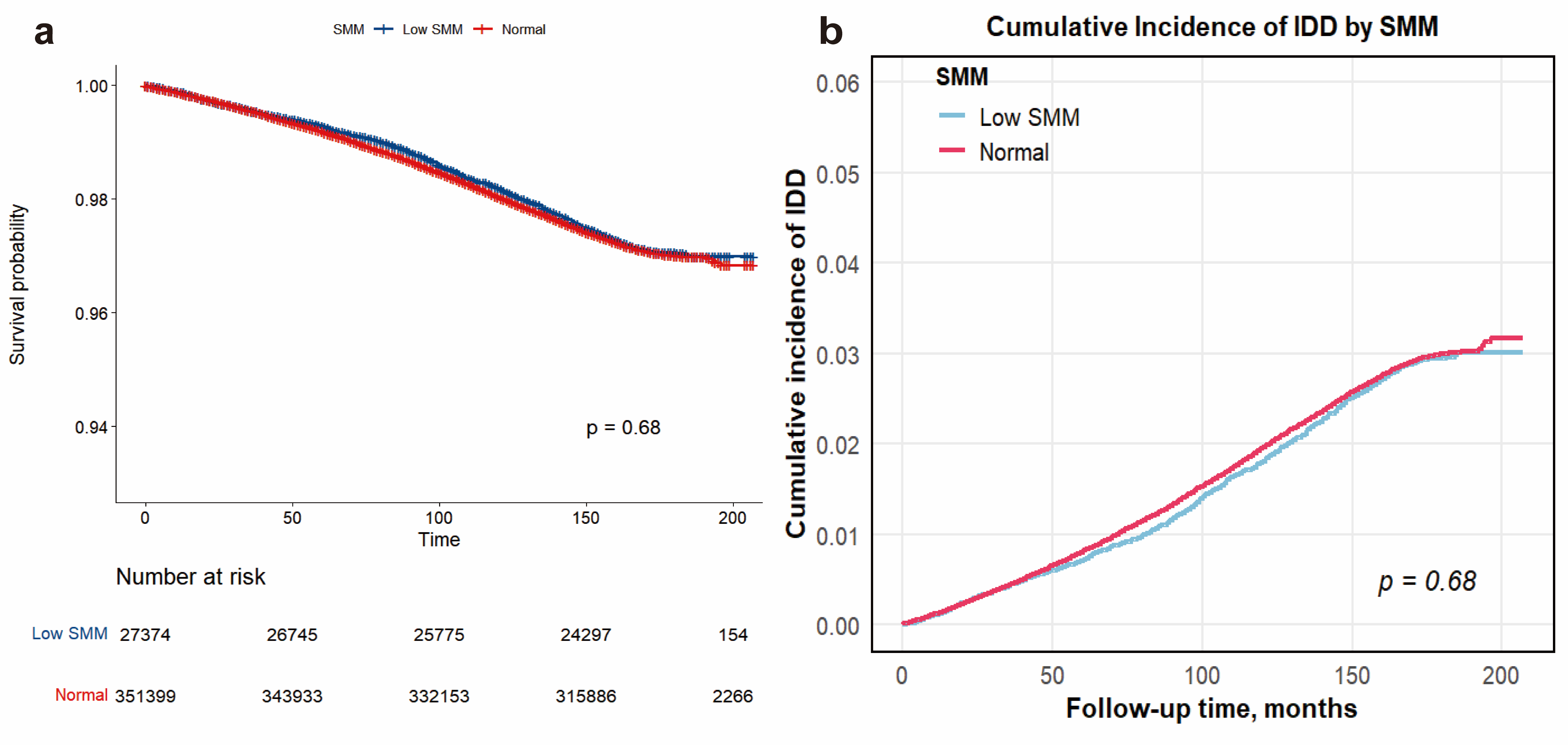

Supplement: Supplementary file 3 — Figure S3: Results of the association analysis between grip strength and the risk of IDD occurrence. Model 1 adjusted for gender, age and BMI. Model 2 adjusted for gender, age, BMI, Townsend deprivation index, education level, smoking, alcohol consumption, physical activity time, sedentary time, history of using corticosteroids, statins and calcium supplements. Model 3: in addition to adjusting for the above variables, also adjusted for heart failure, diabetes, hyperlipidaemia, hypertension and liver disease. GS: Grip strength; IDD: Intervertebral disc degeneration. [file JCSM-16-e70086-s002.tif]

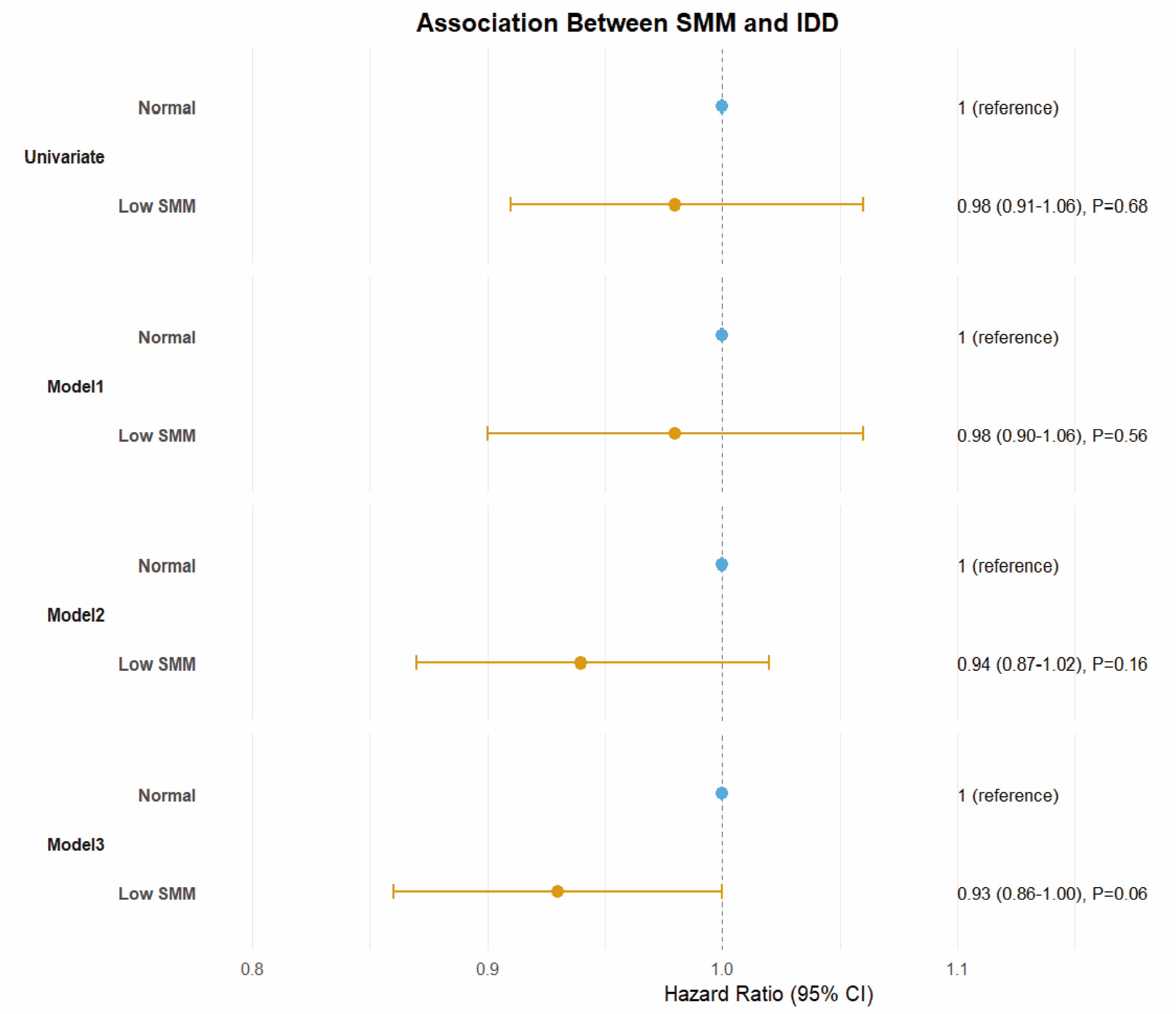

Supplement: Supplementary file 4 — Figure S4: Results of the association analysis between SMM and the risk of IDD occurrence. Model 1 adjusted for gender, age and BMI. Model 2 adjusted for gender, age, BMI, Townsend deprivation index, education level, smoking, alcohol consumption, physical activity time, sedentary time, history of using corticosteroids, statins and calcium supplements. Model 3: in addition to adjusting for the above variables, also adjusted for heart failure, diabetes, hyperlipidaemia, hypertension and liver disease. SMM: skeletal muscle mass; IDD: Intervertebral disc degeneration. [file JCSM-16-e70086-s001.tif]
