# Supplementary material for: Association Between Sarcopenia and the Long‐Term Risk of Intervertebral Disc Degeneration
Source: J Cachexia Sarcopenia Muscle. 2025 Oct 14;16(5):e70086. doi: 10.1002/jcsm.70086 (PMC12519499; doi:10.1002/jcsm.70086)
Supplement: Supplementary file 5 — Data S1: Supplementary references. [file JCSM-16-e70086-s003.docx]

**Supplementary references**

S1. Janssen I, Shepard DS, Katzmarzyk PT, Roubenoff R. The healthcare costs of sarcopenia in the United States. J Am Geriatr Soc. 2004;52(1):80-5 <https://doi.org/10.1111/j.1532-5415.2004.52014.x>

S2. Chengkai L, Junhong L, Zhengya Z, Jiaxiang Z, Fuan W, Fuxin W, et al. Association between grip strength and walking pace with incidence of degenerative cervical myelopathy: a UK biobank observational study. Eur Spine J. 2024<https://doi.org/10.1007/s00586-024-08374-8>

S3. Attaway AH, Lopez R, Welch N, Bellar A, Hatipoğlu U, Zein J, et al. Muscle loss phenotype in COPD is associated with adverse outcomes in the UK Biobank. BMC Pulm Med. 2024;24(1):186 <https://doi.org/10.1186/s12890-024-02999-7>

S4. Elliott AD, Linz D, Mishima R, Kadhim K, Gallagher C, Middeldorp ME, et al. Association between physical activity and risk of incident arrhythmias in 402 406 individuals: evidence from the UK Biobank cohort. European Heart Journal. 2020;41(15):1479-86 <https://doi.org/10.1093/eurheartj/ehz897>

S5. Luo Y, Li Y, Xie J, Duan Y, Gan G, Zhou Y, et al. Symptoms of depression are related to sedentary behavior and sleep duration in elderly individuals: A cross-sectional study of 49,317 older Chinese adults. J Affect Disord. 2022;308:407-12 <https://doi.org/10.1016/j.jad.2022.04.102>

S6. Lv X, Li Y, Li R, Guan X, Li L, Li J, et al. Relationships of sleep traits with prostate cancer risk: A prospective study of 213,999 UK Biobank participants. Prostate. 2022;82(9):984-92 <https://doi.org/10.1002/pros.24345>

S7. Petrosyan E, Fares J, Lesniak MS, Koski TR, El Tecle NE. Biological principles of adult degenerative scoliosis. Trends Mol Med. 2023;29(9):740-52 <https://doi.org/10.1016/j.molmed.2023.05.012>

S8. Chen MJ, Lo YS, Lin CY, Tseng C, Hsiao PH, Lai CY, et al. Impact of sarcopenia on outcomes following lumbar spine surgery for degenerative disease: an updated systematic review and meta-analysis. Eur Spine J. 2024;33(9):3369-80 <https://doi.org/10.1007/s00586-024-08364-w>

S9. Keller K, Engelhardt M. Strength and muscle mass loss with aging process. Age and strength loss. Muscles, ligaments and tendons journal. 2013;3(4):346

S10. Burton LA, Sumukadas D. Optimal management of sarcopenia. Clinical interventions in aging. 2010:217-28

S11. Roh E, Choi KM. Health Consequences of Sarcopenic Obesity: A Narrative Review. Front Endocrinol (Lausanne). 2020;11:332 <https://doi.org/10.3389/fendo.2020.00332>

S12. Tournadre A, Vial G, Capel F, Soubrier M, Boirie Y. Sarcopenia. Joint Bone Spine. 2019;86(3):309-14 <https://doi.org/https://doi.org/10.1016/j.jbspin.2018.08.001>

S13. Haren MT, Siddiqui AM, Armbrecht HJ, Kevorkian RT, Kim MJ, Haas MJ, et al. Testosterone modulates gene expression pathways regulating nutrient accumulation, glucose metabolism and protein turnover in mouse skeletal muscle. International Journal of Andrology. 2011;34(1):55-68 <https://doi.org/https://doi.org/10.1111/j.1365-2605.2010.01061.x>
